# Development and Psychometric Evaluation of Two Scales Assessing Food Allergy Attitudes and Practices Among Food and Beverage Employees: A Preliminary Study

**DOI:** 10.3390/foods15213824

**Published:** 2026-10-26

**Authors:** Hüseyin Gonce, Hasret Gülüş Oymak, Berrak Dumlupınar, Arda Öztürkcan

**Affiliations:** 1 Department of Nutrition and Dietetics, Faculty of Health Sciences, İstanbul Okan University, Istanbul 34959, Türkiye; huseyingonce@gmail.com (H.G.); hasret.oymak@okan.edu.tr (H.G.O.); 2 Department of Biochemistry, Faculty of Pharmacy, Istanbul Okan University, Tuzla, Istanbul 34959, Türkiye; 3 Department of Nutrition and Dietetics, Faculty of Health Sciences, İstanbul Gelişim University, Istanbul 34310, Türkiye; sozturkcan@gelisim.edu.tr

**Keywords:** food allergy, food and beverage employees, attitude, practice, Istanbul, allergen management

## Abstract

This study aimed to develop two instruments assessing food allergy attitudes and practices among food and beverage employees and to evaluate their psychometric properties. A methodological design was used with 110 employees of a hotel food and beverage department in Beyoğlu, İstanbul. Items were generated from the literature and revised based on expert opinion. Construct validity was examined using exploratory factor analysis (EFA), and reliability using Cronbach’s alpha and item–total correlations. The 13-item Attitude Towards Food Allergies Scale showed a four-factor structure explaining 69.18% of the variance (KMO = 0.787; α = 0.811). The 12-item Food Allergy Practices Scale showed a three-factor structure explaining 65.29% of the variance (KMO = 0.818; α = 0.818). Some subscales consisting of two or three items showed low internal consistency and should be interpreted with caution. Mean scores were 40.19 ± 6.43 (attitude; range 0–52) and 21.29 ± 3.47 (practice; range 0–24). Attitude and practice scores were moderately and positively correlated (r = 0.436, *p* < 0.001). Both scales demonstrated acceptable preliminary validity and reliability for assessing food allergy attitudes and practices in food service settings; confirmatory studies in larger and more diverse samples are recommended.

## 1. Introduction

Knowledge, attitudes, and practices of food service professionals regarding food allergies have become pivotal in preventing allergic reactions, especially as prevalence rises. Currently, avoiding offending allergens remains the key prevention method. Insufficient staff training increases the risk of fatal allergic reactions, highlighting the need for effective allergen control and education. Effective staff practices are crucial for allergen control, as lack of knowledge creates challenges for allergic customers. Thus, prioritizing allergen management and comprehensive staff training is vital.

In order to prevent allergic reactions, food service establishments must implement rigorous hygiene practices and staff training targeting allergen management. Studies have shown that systematic approaches and trained personnel are paramount in reducing food allergy risks. To prevent cross-contamination, food preparation areas should be designated separately from serving and cooking areas. For example, frying oils should not be used for non-allergenic foods [1,2,3].

Written standard operating procedures (SOPs) on hygiene and allergen control should be prepared and employee compliance regularly monitored. Clear allergen labeling on all foods and beverages and menu disclosure are essential to inform customers [4].

Therefore, educating employees about food allergies and improving their attitudes and practices are crucial to ensuring customer safety. A systematic approach is also necessary to handle guest allergy inquiries effectively.

Sharma indicated that a considerable proportion of staff exhibited misconceptions regarding allergens. A paucity of training in allergen management may result in legal complications and pecuniary losses for businesses. Therefore, training programmes designed to enhance the knowledge and skills of food service workers are paramount for ensuring customer safety. However, implementation is often hindered by financial constraints, high staff turnover, language barriers, time constraints, and employee disinterest, leading to significant gaps in employees’ knowledge and attitudes [5].

Consequently, enhancing the knowledge, attitudes, and practices of personnel in the food and beverage sector is crucial to preventing allergic reactions and ensuring safety. Employees must undergo regular and mandatory training on hygiene and allergen control, emphasizing: prevention of cross-contact; correct handwashing techniques; equipment cleaning; and allergen management.

Clear procedures should be established to separate allergens at all stages—from storage to processing, transportation, and serving. Suppliers require regular audits, and labeling accuracy must be routinely checked to mitigate allergen contamination risks. Checklists and daily inspections are also essential components of the programme [4,6,7].

Cleaning products should be used according to stipulated instructions, taking into account cleaning time, temperature, and mechanical force. Regular disinfection of high-contact areas and refrigerators is necessary, along with rigorous testing to ensure equipment and surfaces are free from residual protein post-cleaning [8].

To minimize contamination risks, equipment used for allergen-containing and allergen-free foods (e.g., knives, cutting boards, spoons, tongs) should be color-coded and separated. The use of separate baskets for washing equipment or dishwashers is strongly advised [9,10].

Although research has been conducted on the subject of food allergies among restaurant employees in Istanbul [7], there is a paucity of research on this topic in Turkey, particularly given the country’s diverse culinary culture and large food service sector. The attitudes and everyday practices of kitchen and service staff play a pivotal role in preventing allergic reactions, since strict avoidance of the offending allergen remains the primary means of protection for individuals afflicted with food allergies [1,2]. Training is imperative in this regard; however, it must be precise, concise, and readily implementable in hectic work environments. Otherwise, its efficacy diminishes [5,6]. Poor communication between staff members and insufficient training have been identified as significant contributing factors to the risk of cross-contact, particularly in large establishments where responsibilities are distributed across multiple departments and levels of management [2,5]. As demonstrated in previous research, attitudes and practices regarding food allergens are closely interrelated (3). In order to minimise errors in allergen management, it is necessary to implement training programmes that are tailored to the needs of employees, in conjunction with clear communication procedures [2,4,6].

Although food allergy knowledge, attitudes, and practices of food handlers have been examined in previous studies, to our knowledge, no instrument with established psychometric properties is available for assessing the attitudes and practices of food and beverage employees in Türkiye. A valid and reliable instrument is needed to identify training needs and to evaluate the effectiveness of allergen management training in this sector.

The primary aim of this study is to develop two instruments assessing the attitudes and practices of food and beverage employees regarding food allergies and to evaluate their psychometric properties. A secondary aim is to examine the relationship between attitudes and practices. It was hypothesized that attitude scores would be positively correlated with practice scores.

## 2. Materials and Methods

### 2.1. Research Model

This study was conducted within the framework of a methodological research design. Methodological research encompasses studies aimed at developing measurement instruments and evaluating their psychometric properties-specifically validity and reliability [11]. Since the objective of this study was to examine the validity and reliability of the data collection instrument, the methodological research design was deemed appropriate for the study’s purpose. The literature was consulted and expert opinions were obtained during the development process of the measurement instrument.

### 2.2. Study Group

The sample size was determined using the formula $n=\frac{{Nt}^{2}pq}{d^{2}(N\;-\;1)\;+\;t^{2}pq}$ recommended by Yazıcıoğlu and Erdoğan. Assuming a population size of approximately 150 individuals, the minimum required sample size was calculated as 108 at a 95% confidence level and a 5% margin of error. In order to compensate for potential data loss, the target sample size was increased to 110. The study encompassed a total of 110 employees, of whom 65.5% were male and 34.5% were female [12]. The study population consisted of approximately 150 employees working in the food and beverage department of a private members’ club and hotel in the Beyoğlu district of İstanbul. The participant-to-item ratio was approximately 8.5:1 for the Attitude Towards Food Allergies Scale and 9.2:1 for the Food Allergy Practices Scale, meeting the commonly recommended minimum of five participants per item for exploratory factor analysis. Although the data were collected from a single establishment, the sample included employees from different positions (service, kitchen, and managerial staff), age groups, educational levels, and levels of professional experience.

### 2.3. Data Collection

The questionnaire used in the study was developed by adapting items on food allergy attitudes and practices from previous studies [7,13–17]. To ensure the content validity of the measurement tool, the opinions of experts experienced in quality management and quality/hygiene auditing within the food and beverage sector were sought, and the items were revised in accordance with their evaluations. Accordingly, three distinct measurement tools were developed for the study—Attitude Towards Food Allergies Scale and Food Allergy Practices Scale-and their validity and reliability characteristics were assessed through psychometric analyses [7,13,14,15,16,17].

#### 2.3.1. Demographical Information Form

The Demographic Information Form comprised 13 questions concerning participants’ sociodemographic characteristics, hygiene training, allergen-related procedures, and food allergies. The form was developed by the researchers for the purpose of evaluating the professional and personal profiles of the participants, in addition to their experience with regard to food allergies. In addition to providing a detailed description of the characteristics of the study sample, the information obtained contributed to the evaluation of potential associations between sociodemographic variables and participants’ levels of knowledge, attitudes, and practices.

#### 2.3.2. Attitude Towards Food Allergies Scale

This scale was developed to assess participants’ attitudes toward food allergies. The measurement tool consists of a total of 13 items. Participants were asked to respond to each item using a 5-point Likert-type scale ranging from “Strongly Disagree” to “Strongly Agree.” Responses were scored from 0 to 4 points, resulting in a total attitude score ranging from 0 to 52. Items 2 and 6 were reverse-coded prior to analysis so that a high score indicates greater safe and appropriate practices. A higher total score indicates a more positive attitude toward food allergies, whereas a lower score indicates a more negative attitude.

#### 2.3.3. Development of the Food Allergy Practices Scale

This scale was developed to assess participants’ levels of practice and behavior regarding food allergies. Accordingly, the questionnaire includes a section on practices consisting of 12 items. Participants were asked to respond to statements regarding these practices using a 3-point Likert-type scale with the options “Never,” “Sometimes,” and “Always.” Responses were scored as 0, 1, and 2 points, respectively, resulting in a total possible score ranging from 0 to 24. Item 9 was reverse-coded prior to analysis so that a high score indicates safer and more appropriate practices. Higher total scores indicate safer and more appropriate practices.

### 2.4. Statistical Analysis

The SPSS 26.0 statistical software was used to analyze the research data. Prior to analysis, the dataset was examined for outliers and extreme values, and none were found. Exploratory factor analysis (EFA) was conducted to assess the construct validity of the measurement instruments, while Cronbach’s alpha coefficients and item-total correlations were examined to evaluate their reliability. Additionally, descriptive statistics were calculated to determine the general characteristics of the data, and the normality of the distribution was assessed using skewness and kurtosis values. Based on the findings, it was determined that the data followed a normal distribution, and Pearson correlation analysis was performed to examine the relationships between the variables.

## 3. Results

In this section, findings regarding the analysis of data obtained within the scope of the research are presented, and the results derived from the validity and reliability analyses of the measurement instruments are reported.

### 3.1. General Characteristics of the Participants

The study group consisted of a total of 110 employees working in the food and beverage department of a hotel operating in the Beyoğlu district of Istanbul. Regarding the gender distribution of the participants, 38 participants (34.5%) were female and 72 (65.5%) were male. In terms of age distribution, 36 participants (32.7%) were aged 18–25 years, 46 (41.8%) were aged 26–33 years, and 28 (25.5%) were aged 34 years or older. Regarding educational level, 50 participants (45.5%) had a high school education or lower, while 60 participants (54.5%) had an associate degree or higher. Regarding their positions in the establishment, 51 participants (46.4%) worked in service (including managerial service staff), 47 (42.7%) worked in the kitchen (including managerial chefs and dishwashers), and 12 (10.9%) held other positions. In terms of work experience in food and beverage establishments, 48 participants (43.6%) had less than 6 years of experience, 32 (29.1%) had 6–10 years, and 30 (27.3%) had 11 years or more of experience. It was found that 98 participants (89.1%) had a hygiene certificate, whereas 12 (10.9%) did not. Regarding whether an allergic reaction had previously occurred in the restaurant where they worked, 13 participants (11.8%) answered yes, while 97 (88.2%) answered no. In terms of whether the establishment had a written allergen procedure, 87 participants (79.1%) reported that such a procedure existed, whereas 23 (20.9%) reported that it did not. It was determined that 12 participants (10.9%) had a food allergy, while 98 (89.1%) did not. Regarding whether they knew someone with a food allergy, 43 participants (39.1%) answered yes and 67 (60.9%) answered no. In terms of allergen training, 73 participants (66.4%) had received training, whereas 37 (33.6%) had not. When practices related to allergens were evaluated, 63 participants (57.3%) reported experience or practice related to cross-contact, 54 (49.1%) related to communication with customers, 66 (60.0%) related to identifying allergens in menu items, 47 (42.7%) related to recognizing symptoms of an allergic reaction, 61 (55.5%) related to reading/understanding food ingredients, and 33 (30.0%) related to managing an allergic reaction. The sociodemographic characteristics of the participants included in the study are presented in Table 1.

### 3.2. Reliability Coefficients of Attitude Towards Food Allergies Scale

An exploratory factor analysis (EFA) was conducted to examine the factor structure of the Attitude Towards Food Allergies Scale. The scale was found to have a four-factor structure (Table 2). The Kaiser–Meyer–Olkin (KMO) measure of sampling adequacy was 0.787, and Bartlett’s test of sphericity was statistically significant (χ^2^ = 587.996; *p* < 0.001). These findings indicate that the data were suitable for factor analysis.

**Table 2:** Factor loadings and reliability coefficients of attitude towards food allergies scale.

Scale Items	1. Factor	2. Factor	3. Factor	4. Factor	Item-Total Correlation
1. I believe that all employees should be able to accurately identify the ingredients in the food served.	0.67				0.343
2. I believe that food and beverage industry employees (chefs, service staff, managers, or cashiers) should have knowledge about food allergies.	0.67				0.641
3. I believe that food allergies should be taken very seriously in the restaurant industry.	0.85				0.669
4. I believe that restaurant staff should be regularly informed and receive periodic training about food allergies.	0.82				0.517
5. I believe that safe food can be prepared for customers with food allergies.			0.74		0.447
6. I believe that appropriate measures can be taken in restaurants to prevent cross-contact between foods.			0.82		0.296
7. I believe that receiving services related to food allergies in restaurants is everyone’s right.		0.54			0.600
8. I believe that producing food safely for customers with food allergies is our responsibility toward them.		0.76			0.504
9. I believe that preventing food allergy incidents in restaurants is one of the responsibilities of the establishment.			0.59		0.606
10. In my opinion, a customer who reports having a food allergy should not be turned away.		0.79			0.424
11. I believe that people with food allergies should also be able to eat at restaurants.				0.60	0.466
12. I believe that alternative food preparation and serving options should be provided to customers who report having a food allergy to meet their dietary needs.		0.74			0.610
13. I believe that serving customers with food allergies in a food and beverage establishment is not difficult.				0.85	0.307
Kaiser–Meyer–Olkin: 0.787 Bartlett’s Test Chi-Square Value: 587.996 Bartlett’s Test Significance Coefficient: *p* < 0.001					
Variance Explained by Each Factor	22.77%	20.04%	15.96%	10.40%	
Cronbach Alpha Coefficient by Each Factor	0.830	0.771	0.653	0.404	
Total Variance Explained	69.18%				
Cronbach Alpha (Total Score)	0.811				

The four factors explained 69.18% of the total variance. The first factor accounted for 22.77%, the second factor for 20.04%, the third factor for 15.96%, and the fourth factor for 10.40% of the total variance. Examination of the factor loadings showed that the items loading on the first factor (Items 1, 2, 3, and 4) had factor loadings ranging from 0.67 to 0.85. The factor loadings of the items loading on the second factor (Items 5, 6, 8, and 9) ranged from 0.59 to 0.82. The items loading on the third factor (Items 7, 10, and 12) had factor loadings ranging from 0.54 to 0.79. Finally, the factor loadings of the items loading on the fourth factor (Items 11 and 13) ranged from 0.60 to 0.85. These findings indicate that the items were sufficiently associated with their respective factors.

Item-total correlations ranged from 0.296 to 0.669. The majority of the items showed acceptable item-total correlations, suggesting consistent relationships with the overall scale score. Items 6 (r = 0.296) and 13 (r = 0.307), however, had relatively lower item-total correlations compared to other items. Despite this, these items were retained in the scale because they exhibited adequate factor loadings, and the overall internal consistency of the scale remained satisfactory. This approach balances item retention with maintaining the scale’s reliability and construct validity.

The reliability of the scale was evaluated using Cronbach’s alpha coefficients, with the total scale achieving a Cronbach’s alpha of 0.811. The internal consistency coefficients for the four factors were 0.830, 0.771, 0.653, and 0.404, respectively. The first factor demonstrated high internal consistency, while the second factor showed acceptable reliability. The reliability coefficient of the third factor was relatively low but can be considered marginally acceptable. The fourth factor demonstrated low internal consistency (α = 0.404). This relatively low coefficient may be partly attributable to the fact that the fourth factor consists of only two items, as Cronbach’s alpha is sensitive to the number of items within a factor.

Based on the content of the items, the first factor includes statements concerning employees’ knowledge and awareness of food allergies, the accurate identification of food ingredients, taking food allergies seriously, and providing regular information and training to staff. Therefore, this factor can be labeled “Food Allergy Knowledge and Awareness.” The second factor consists of items related to preparing safe food for customers with food allergies, taking appropriate measures to prevent cross-contact, ensuring safe food production, and recognizing the responsibility of food establishments in preventing food allergy incidents. Accordingly, this factor can be labeled “Safe Food Preparation and Responsibility.” The third factor includes items concerning the right of customers with food allergies to receive restaurant services, not turning away customers who report having a food allergy, and providing alternative food preparation and serving options to meet their dietary needs. Therefore, this factor can be labeled “Customer Rights and Inclusive Service.” The fourth factor consists of items concerning the feasibility of serving customers with food allergies and their ability to eat at restaurants. Thus, this factor can be labeled “Ease of Serving Customers with Food Allergies.”

Overall, the findings indicate that the Attitude Towards Food Allergies Scale has a four-factor structure explaining 69.18% of the total variance, with generally adequate factor loadings and satisfactory overall internal consistency (Cronbach’s α = 0.811). The findings indicate that the scale demonstrates good construct validity and overall reliability. Nevertheless, the relatively low internal consistency coefficient reported for the fourth factor suggests that interpretations based on this subscale should be approached with caution. To enhance the reliability of this factor, future research might consider incorporating additional items, which could strengthen the internal consistency and improve the measurement precision of this subdimension.

### 3.3. Food Allergy Practices Scale

An exploratory factor analysis (EFA) was conducted to examine the factor structure of the Food Allergy Practices Scale. The scale was found to have a three-factor structure (Table 3). The Kaiser–Meyer–Olkin (KMO) measure of sampling adequacy was 0.818, and Bartlett’s test of sphericity was statistically significant (χ^2^ = 658.991; *p* < 0.001). These findings indicate that the data were suitable for factor analysis.

**Table 3:** Factor loadings and reliability coefficients of food allergy practices scale.

Scale Items	1. Factor	2. Factor	3. Factor	Item-Total Correlation
1. I communicate with all my team members (kitchen & service) to ensure that food is prepared safely and is allergen-free when one of my customers has a food allergy.		0.71		0.361
2. I listen carefully to customers, understand their concerns, and then respond to them regarding food allergens in meals.	0.92			0.675
3. If a mistake is made while preparing a meal for a customer with a food allergy, I remake the meal.	0.89			0.755
4. I use clean and sanitized equipment and utensils at my workplace to prevent cross-contact of allergens between foods.	0.70			0.667
5. Before preparing/serving an allergen-free food/beverage, I thoroughly wash my hands with soap and water and put on a new pair of gloves.	0.76			0.370
6. When a customer identifies themselves as having a food allergy, I provide them with accurate information about the ingredients and preparation methods.	0.75			0.597
7. I use separate equipment (tongs, spoons) to handle foods containing allergens.		0.83		0.439
8. When using packaged food, I read the label and check for allergens before preparing and serving the product.		0.49		0.640
9. When serving customers with food allergies, I keep trays containing allergenic and non-allergenic foods separate to prevent cross-contact.	0.66		0.	0.784
10. If I am unsure about the ingredients of a menu item, I do not tell the customer that the food does not contain any allergens.			0.68	0.298
11. When preparing deep-fried food for customers with food allergies, I make sure to change the oil in the fryer to prevent cross-contact.	0.54			0.691
12. When preparing food for a customer with a food allergy, I pay greater attention to safe food practices than I do when preparing food for customers without food allergies.			0.75	0.381
Kaiser–Meyer–Olkin: 0.818 Bartlett’s Test Chi-Square Value: 658.991 Bartlett’s Test Significance Coefficient: *p* < 0.001				
Variance Explained by Each Factor	35.52%	19.43%	10.32%	
Cronbach Alpha Coefficient by Each Factor	0.854	0.578	0.367	
Total Variance Explained	65.29%			
Cronbach Alpha (Total Score)	0.818			

The three factors explained 65.29% of the total variance. The first factor accounted for 35.52%, the second factor for 19.43%, and the third factor for 10.32% of the total variance. Examination of the factor loadings showed that the items loading on the first factor (Items 2, 3, 4, 5, 6, 9, and 11) had factor loadings ranging from 0.54 to 0.92. The factor loadings of the items loading on the second factor (Items 1, 7, and 8) ranged from 0.49 to 0.83. The items loading on the third factor (Items 10 and 12) had factor loadings of 0.68 and 0.75, respectively. These findings indicate that the items were sufficiently associated with their respective factors.

Item-total correlations ranged from 0.298 to 0.784. The majority of the scale items showed acceptable item-total correlations, suggesting that they were consistently related to the overall scale score. Notably, Item 9 had the highest item-total correlation (r = 0.784), indicating a strong contribution to the scale, while Item 10 exhibited the lowest correlation (r = 0.298). These item-total correlation values imply that, overall, the items adequately contributed to measuring food allergy practices, supporting the scale’s construct validity and reliability.

The reliability of the scale was evaluated using Cronbach’s alpha coefficients. The total scale exhibited a Cronbach’s alpha of 0.818. The internal consistency coefficients of the three factors were 0.854, 0.578, and 0.367, respectively. While the first factor showed good internal consistency, the coefficients of the second and third factors were below the commonly accepted threshold of 0.70. Because a satisfactory total alpha does not guarantee the reliability of individual subscales in a multidimensional scale, these findings indicate that the second and third factors may not reliably measure their intended constructs.

Based on the content of the items, the first factor includes practices related to communicating with team members to ensure allergen-free food preparation, carefully responding to customers’ concerns about food allergens, remaking meals when an error occurs, using clean and sanitized equipment, maintaining appropriate hand hygiene and glove use, providing accurate information about ingredients and preparation methods, keeping allergenic and non-allergenic foods separate, and changing fryer oil to prevent cross-contact. Therefore, this factor can be labeled “Safe Food Preparation and Cross-Contact Prevention.”

The second factor includes practices concerning communication and the use of separate equipment when handling allergenic foods, as well as checking packaged food labels for allergens before preparation and service. Accordingly, this factor can be labeled “Allergen Identification and Handling Practices.”

The third factor consists of practices related to avoiding unsupported claims about the absence of allergens when the ingredients of a menu item are uncertain and paying greater attention to safe food practices when preparing food for customers with food allergies. Therefore, this factor can be labeled “Cautious and Enhanced Allergy-Safe Practices.”

Overall, the findings indicate that the Food Allergy Practices Scale has a three-factor structure explaining 65.29% of the total variance, with factor loadings ranging from 0.49 to 0.92 and satisfactory internal consistency for the total scale (Cronbach’s α = 0.818). However, the low internal consistency of the second and third factors indicates that, at this stage, only the total score and the first factor can be considered reliable, and the subscale structure of the scale should be regarded as preliminary.

### 3.4. Attitude and Practice Scores of the Participants

The mean score of the Attitude Towards Food Allergies Scale was 40.19 ± 6.43 (possible range: 0–52), corresponding to 77.3% of the maximum possible score, and the mean score of the Food Allergy Practices Scale was 21.29 ± 3.47 (possible range: 0–24), corresponding to 88.7% of the maximum possible score (Table 4). According to George and Mallery, skewness and kurtosis values between −2 and +2 indicate that the data conform to a normal distribution [18]. As shown in Table 4, the skewness and kurtosis values of both scale scores fell within this range; therefore, the data were considered normally distributed.

#### Pearson Correlation Analysis Results

A statistically significant, moderate positive correlation was found between the total score of the Food Allergy Practices Scale and the total score of the Attitude Towards Food Allergies Scale (r = 0.436, *p* < 0.001) (Table 5). This finding indicates that higher levels of food allergy practices were associated with more positive attitudes toward food allergies.

**Table 5:** Correlation between scale scores.

		Food Allergy Practices Scale (Total)
Attitude Towards Food Allergies Scale (Total)	r	0.436
	*p*	<0.001

## 4. Discussion

Due to their increasing prevalence, food allergies have become a significant issue posing a threat to global public health. This situation holds particular importance within the food service sector. The degree of knowledge, attitudes, and practices exhibited by personnel operating within the food and beverage sector concerning food allergies is of paramount importance in ensuring consumer health protection and fulfilling the legal obligations of businesses. In this context, research provides a scientific basis for the development of effective intervention and training programmes by revealing the current situation in the sector [19,20,21].

The present study was designed in response to this need and aims to develop and evaluate instruments measuring the attitudes and practices of employees in the food and beverage sector regarding food allergies in a multidimensional way. This study makes significant contributions to the existing literature in terms of sample structure and the data collection method. This study offers contemporary and practical insights aimed at addressing existing knowledge gaps in the field [6,7]. Moreover, deficiencies in staff training and insufficient knowledge levels regarding food allergies are recognized as global challenges in the international literature, with frequent emphasis on sectoral workforce turnover and the difficulties in maintaining continuous education [22,23,24]. In this study, two instruments were developed to assess the attitudes and practices of food and beverage employees regarding food allergies. The EFA results supported a four-factor structure for the Attitude Towards Food Allergies Scale, explaining 69.18% of the total variance, and a three-factor structure for the Food Allergy Practices Scale, explaining 65.29% of the total variance. The factor loadings of both scales (0.54–0.85 and 0.49–0.92, respectively) exceeded the commonly accepted threshold of 0.40, and the KMO values (0.787 and 0.818) together with significant Bartlett’s tests indicated that the data were suitable for factor analysis despite the relatively small sample size. The Cronbach’s alpha coefficients for the total scales (0.811 and 0.818) exceeded the recommended threshold of 0.70, indicating satisfactory internal consistency. These findings support the use of the total scores of both scales for assessing food allergy attitudes and practices among food and beverage employees.

However, some subscales showed low internal consistency, particularly the fourth factor of the attitude scale (α = 0.404) and the second and third factors of the practice scale (α = 0.578 and α = 0.367, respectively). Cronbach’s alpha is strongly influenced by the number of items, and these subscales consisted of only two or three items. Furthermore, the items within these factors may reflect behaviours that are interrelated but conceptually distinct; for example, avoiding unsubstantiated claims regarding allergen content and taking extra care when preparing food for customers with food allergies. The three-point response format of the application scale also limits response variance, which may further reduce reliability estimates. Consequently, subscale scores should be interpreted with caution, and it is recommended that total scores be used at this stage. It is recommended that subsequent studies contemplate the incorporation of novel items to these subscales or the evaluation of alternative factor solutions. The mean application score (21.29 ± 3.47 out of 24) corresponds to approximately 89 per cent of the maximum possible score, which indicates a possible ceiling effect. This situation may limit the scale’s ability to distinguish between employees with high and very high levels of safe practice and to identify improvements following training interventions. Given that the assessments are based on employees’ self-reports, it is possible that social desirability bias may also have influenced the high scores obtained. The fact that a significant proportion of participants held a hygiene certificate (89.1 per cent) and stated that their businesses had a written allergen procedure in place (79.1 per cent) may provide a partial explanation for these findings. In subsequent studies, the utilisation of observational methods for the validation of self-reported practices should be contemplated.

The moderate positive correlation between attitude and behaviour scores (r = 0.436, *p* < 0.001) is consistent with the theoretical expectation that attitudes are related to behaviour, and provides preliminary evidence supporting the construct validity of the scales. Moreover, the moderate strength of this correlation suggests that the two scales in question are related but measure different constructs.

Accordingly, there is a pronounced need for effective and sustainable training models at both local and global scales [5,21,23]. By integrating perspectives from both local contexts and the broader international discourse, this research makes a significant contribution to the management of food allergies.

The majority of participants were in the 26–33 age group (41.8%), and male employees outnumbered female employees (65.5% vs. 34.5%). Slightly more than half of the participants (54.5%) held an associate degree or higher, and 43.6% had less than six years of experience in the food and beverage sector. Most participants held a hygiene certificate (89.1%) and reported that a written allergen procedure existed in their establishment (79.1%), whereas one-third (33.6%) had not received any allergen training. These sample characteristics should be taken into account when interpreting the findings, and training programmes aimed at improving food allergy management should be designed with consideration for the diverse demographic and professional profiles of employees.

The high proportion of participants holding a hygiene certificate (89.1%) suggests that hygiene and food safety are prioritized in the establishment. In addition, 88.2% of participants reported that no allergic reaction had occurred in their workplace. Additionally, studies conducted in European countries and other regions reveal that employees receive inadequate training in allergen identification, cross-contamination prevention, and emergency management [25]. In the global literature, a plethora of training programmes have been proposed and implemented to address the risks posed by knowledge gaps regarding food allergies. For instance, the food allergy training modules developed by the Food Standards Agency in the UK have been shown to serve as a model with regard to increasing awareness and disseminating safe practices in the sector [26].

In addition, within the framework of social learning theory, Bandura’s self-efficacy approach emphasises the pivotal role of the conversion of knowledge and competencies into tangible actions. Effective training programmes have been shown to enhance performance by increasing motivation and self-confidence [2,5,7,13,15,27].

Within this international framework, the expansion and assurance of the continuity of analogous training programmes in Türkiye would constitute significant steps in addressing the knowledge and practice disparities within the sector. In conclusion, the challenges faced by employees in the food and beverage sector in relation to allergen knowledge and the development of safe behaviours are universal and can be solved with effective, continuously updated, and practice-oriented training policies. The implementation of these approaches, as evidenced in the extant literature, particularly within the Turkish context, would serve to safeguard consumer health while ensuring full compliance with legal obligations. Nevertheless, other studies have revealed that employees receive inadequate training in allergen identification and emergency response [3,14,20,23,24].

Positive attitudes toward food allergy management were evident, emphasizing the role of employee knowledge and ongoing training as collective organizational responsibilities. A significant positive correlation between attitudes and practices was observed, with participants exhibiting conscientious behaviors in communication, hygiene, and equipment use. Elevated rates of label reading and allergen awareness, along with recognition of improper hygiene practices (e.g., allergen removal, handwashing, glove use), further support the association between attitudes and practices. These findings reinforce the critical role of effective training programs in fostering awareness and promoting safe allergen management within food service settings [7,13,14,15,17,28]. At the item level, the lowest average score on the Food Allergy Practices Scale was recorded for item 10, which relates to the failure to inform customers that a dish of uncertain composition does not contain allergens (average = 1.62 out of 2). While the majority of participants indicated that they adhered to this practice, the findings suggest that some staff members, at least on occasion, provided customers with assurances that could not be substantiated. As was also noted in earlier studies [7,14,28], comparable deficiencies in the domain of customer communication have been reported.

This situation reveals the information gaps and self-efficacy concerns among personnel in the food and beverage sector, emphasizing the importance of accurate and effective communication. From a public health perspective, ensuring the accuracy and reliability of information related to food allergies is critical for preventing emergency health risks. Therefore, training programs should focus not only on the transfer of information but also on enhancing employees’ decision-making competence [2,7,14,21].

In conclusion, while cautious communication is positive, more comprehensive training and monitoring mechanisms are necessary to prevent allergic reactions caused by misinformation and to protect public health.

Demographic differences in attitude and practice scores were not analysed in this study, since our main aim was to develop the two instruments and test their psychometric properties. Earlier research suggests that such differences may exist. Tatlı and Akoğlu found that female restaurant employees knew significantly more about food allergies than their male colleagues [7], and Radke et al. reported a similar pattern among restaurant managers and staff [29]. The question of whether the scales developed here possess sufficient sensitivity to detect such variations remains unresolved. Consequently, a subsequent step would be to test these scales on groups defined by criteria such as gender, educational level, job position or allergen training. This would provide evidence of their validity.

These sample characteristics also have practical implications for educational design. A number of studies have been conducted in Turkey on the subject of allergen training and the outcomes thereof. It has been demonstrated that staff members employed in the restaurant sector who have undergone training in food allergies have been found to exhibit significantly safer practices than their counterparts who have not received such training [7]. Furthermore, Bucak and Yiğit have highlighted the role played by the level of training amongst hotel service and kitchen staff [16]. Staff members who are younger or have received a more limited formal education may have encountered reduced opportunities to acquire knowledge in the domain of allergen management within structured educational settings. Consequently, training materials directed towards these groups should be succinct, focused on key messages, visually engaging, and structured around concrete, everyday tasks such as checking labels, responding to customer queries, or preparing food without cross-contamination. The provision of content at varying language levels has also been demonstrated to facilitate the dissemination of information to staff members with a range of educational backgrounds [19].

Allergen training deserves particular attention in this regard. One-third of the participants (33.6%) reported that they had never received allergen training, even though most held a hygiene certificate (89.1%). Earlier studies have demonstrated the efficacy of this training method. Tatlı and Akoğlu’s research revealed that restaurant staff who had undergone training on food allergies exhibited significantly safer practices in comparison to those who had not received such training [7]. The scales developed in this study have the potential to facilitate a more detailed examination of this relationship, both through the comparison of trained and untrained staff members and by evaluating attitudes and practices before and after a training programme. However, given the high average scores obtained on the practice scale, the scale’s ability to detect post-training improvement may be limited. This should be taken into account in intervention studies.

When all these factors are considered together, it becomes clear that training programmes should be planned taking into account employees’ educational background and prior training, and that the impact of these programmes should be assessed using appropriate measurement tools.

Overall, participants reported generally positive attitudes and largely safe practices regarding food allergies. The mean attitude score corresponded to 77.3% and the mean practice score to 88.7% of the maximum possible score. Table 1, however, points to some gaps. Fewer than half of the participants reported having experience in recognising the symptoms of an allergic reaction (42.7%) or in communicating with customers about allergens (49.1%), and only 30.0% reported experience in managing an allergic reaction.

In the context of the attitude scale, the two items exhibiting the lowest item-total correlations were identified as item 6, pertaining to the belief that appropriate measures can be taken to prevent cross-contamination (r = 0.296), and item 13, concerning the belief that serving customers with food allergies is not a challenging task (r = 0.307). In contrast to the majority of items, which reflect the extent to which staff take food allergies seriously, these two items reflect the extent to which staff find allergen management practicable in practice. This scenario demonstrates that, despite a member of staff’s perception of food allergies as a significant concern, there may be residual apprehension regarding the delivery of safe service in a high-pressure kitchen environment. This perception of feasibility is closely linked to self-efficacy, which is defined as a key link between training and actual behaviour [2,5,7]. Consequently, training programmes may need to go beyond simply raising awareness; for example, they should also help to build staff confidence that allergen-safe service is achievable, through practical exercises and simulations.

It is imperative that food allergy training encompasses the fundamental principles of allergen management. This encompasses the definition of food allergy, the identification of the primary allergens, an analysis of cross-contamination risks, and an examination of hygiene and sanitation procedures [2,4]. Furthermore, it is imperative that staff members are made aware of the potential consequences of certain contributing factors, such as alcohol consumption or physical exercise, which have been observed to exacerbate the severity of allergic reactions. This will ensure that they possess a comprehensive understanding of the risks associated with specific combinations of food and drink. In view of the paucity of experience in recognising and managing allergic reactions in this sample, emergency management should be accorded a key role in training. As demonstrated by the extant literature, structured training programmes have been shown to enhance competence in managing allergic reactions [2,27]. Therefore, it is recommended that such training programmes include the recognition of early signs of anaphylaxis, the knowledge of when to call for emergency assistance, and the use of an adrenaline auto-injector.

In addition to conventional seminar- and brochure-based methods, priority should be given to interactive and practical techniques as part of the training methodology [30]. In light of the challenges encountered within the sector, including a demanding work environment and staff turnover, simulations, case studies, e-learning modules and video-based training have been identified as offering more flexible and effective learning opportunities, thereby enhancing self-efficacy in relation to knowledge. Moreover, the development of educational content tailored to different language proficiency levels has been identified as a key strategy for overcoming language barriers and increasing participation in education [19].

In the context of training for food allergy management, it is imperative that the curriculum is subject to periodic review and refinement. It is imperative that the training content is subject to regular refreshment in accordance with the emergence of new scientific developments, legislative changes and feedback from the workplace. In order to ensure the continuity of these updates, measures such as on-the-job training, professional development courses and certificate renewal processes are recommended. The effectiveness of training programmes should be evaluated through the utilisation of methods such as knowledge assessment tests and practical examinations. Moreover, in order to address knowledge gaps swiftly and enhance staff motivation, it is essential to continuously refine the content and methods in accordance with staff feedback [7].

## 5. Limitations and Future Research

The present study is subject to several limitations. Firstly, the data were collected from a single establishment, namely a private members’ club and hotel in the Beyoğlu district of Istanbul. This limits the generalisability of the findings to the wider food and beverage sector. Secondly, the sample size (n = 110) is relatively small for the scale development process. Although the KMO values indicate sample adequacy, the factor structures need to be validated in larger samples. Thirdly, it should be noted that only exploratory factor analysis was carried out; confirmatory factor analysis was not performed. Therefore, it can be concluded that the factor structures of both scales should be regarded as preliminary. Fourthly, the temporal stability of the scales is unknown due to the absence of an assessment of test–retest reliability. Fifthly, the convergent and discriminant validity of the measurement tools employed has not been examined using other established measurement tools. Finally, the cross-sectional design does not allow for causal inferences regarding the relationship between attitudes and practices. Furthermore, data based on participants’ self-reports may be subject to social desirability bias. It is recommended that future research seeks to validate the factor structures of both scales using confirmatory factor analysis on larger and more diverse samples drawn from different regions and types of organisations. In addition, the test–retest reliability of the scales should be assessed.

## 6. Conclusions

Based on the study findings, training programs should go beyond merely conveying information; they should aim to improve employees’ practical skills and encourage behavioral change. Implementing mandatory, on-the-job training with periodic refreshers is essential to maintain updated employee knowledge, which is critical for effective allergen management and ensuring the sustainability of safe food services. In this context, it is recommended that training modules be structured under the headings of basic food allergy knowledge, emergency management, prevention of cross-contamination, and customer communication. Interactive and hands-on methods, such as virtual simulations and case studies, have been demonstrated to be effective in increasing participants’ knowledge and awareness. Furthermore, the periodic updating and evaluation of training content plays a critical role in ensuring sustainability in allergen management. This finding serves to reinforce the positive correlation between attitudes and practices. Furthermore, the allocation of adequate financial resources and the establishment of sustainable training infrastructures are imperative to ensure the effective and accurate management of demands related to food allergies in the food and beverage industry. Empirical evidence has demonstrated that training methods which are empathetic and participatory will contribute to increasing employees’ awareness of food allergens and developing effective application skills. The study emphasizes the importance of integrating food allergy and emergency management topics into the adult-only ‘Hygiene Training for Food and Water Sector Workers’ program, especially targeting kitchen and service personnel. This inclusion is expected to enhance employees’ awareness of food allergies and improve their ability to respond promptly and effectively to allergic emergencies, thereby increasing customer safety and the overall reliability of the food and beverage sector. Additionally, regularly updating the training content, continuously monitoring workplace practices, and providing expert support are vital measures for sector development and public health protection. Consequently, training programmes should not only facilitate the transfer of information but also be application-oriented and supported by continuously updated content.

## Figures and Tables

**Table 1:** Sociodemographic characteristics of the participants.

Variable	Group	n	%
Gender	Female	38	34.50
	Male	72	65.50
Age	18–25 years	36	32.70
	26–33 years	46	41.80
	34 years and older	28	25.50
Educational Level	High school or below	50	45.50
	Associate degree or higher	60	54.50
Position in the Establishment	Service personnel	40	36.40
	Managerial service personnel	11	10.00
	Chef/Cook	38	34.50
	Managerial chef/Cook	7	6.40
	Dishwasher	2	1.80
	Other	12	10.90
Experience in Food and Beverage	Less than 6 years	48	43.60
Establishments	6–10 years	32	29.10
	11 years or more	30	27.30
Hygiene Certificate	Yes	98	89.10
	No	12	10.90
Previous Allergic Reaction at the Restaurant	Yes	13	11.80
	No	97	88.20
Written Allergen Procedure at the Establishment	Yes	87	79.10
	No	23	20.90
Food Allergy	Yes	12	10.90
	No	98	89.10
Knowing Someone with a Food Allergy	Yes	43	39.10
	No	67	60.90
Allergen Training	Yes	73	66.40
	No	37	33.60
Cross-contact	Yes	63	57.30
	No	47	42.70
Communication with Customers	Yes	54	49.10
	No	56	50.90
Identification of Allergens in Menu Items	Yes	66	60.00
	No	44	40.00
Recognition of Allergic Reaction Symptoms	Yes	47	42.70
	No	63	57.30
Reading/Understanding Food Ingredients	Yes	61	55.50
	No	49	44.50
Management of an Allergic Reaction	Yes	33	30.00
	No	77	70.00

**Table 4:** Descriptive statistics for scale scores.

Variable	Mean	SD	Min.	Max.	Skewness	Kurtosis
Attitude Towards Food Allergies Scale (Total)	40.19	6.43	20	51	−0.803	−1.627
Food Allergy Practices Scale (Total)	21.29	3.47	12	24	0.773	1.605

## Data Availability

The data supporting the findings of this study are available from the corresponding author upon reasonable request. The thesis from which this article was derived will be publicly accessible through the National Thesis Center of the Council of Higher Education (YÖK), Türkiye.
